# Supplementary material for: Are carbon emissions trading and green financial instruments synergistic? -Comprehensive quantitative research based on content analysis
Source: PLoS One. 2024 Mar 7;19(3):e0298601. doi: 10.1371/journal.pone.0298601 (PMC10919601; doi:10.1371/journal.pone.0298601)
Supplement: S1 Data — (ZIP) [file pone.0298601.s001.zip › Data/地级市Z_F工作报告.zip!/╣π╬≈/╣≤╕█╩╨/└┤╫╘╣1⁄2╓┌║┼í╛┬φ┐╦╩2╛▌═°í┐_2009.docx]

各位代表：

现在，我代表市人民政府向大会作工作报告，请各位代表审议，并请各位政协委员和列席会议的同志提出建议和意见。

2008年工作回顾

2008年，我们在自治区党委、政府和市委的正确领导下，在市人大及其常委会、市政协的监督支持下，坚持以邓小平理论和“三个代表”重要思想为指导，以科学发展观统领全局，按照“六靠”工作思路，围绕建设富裕文明和谐贵港的目标，带领和依靠全市人民，努力克服年初低温冰雪、夏季台风和反常冬汛等严重的自然灾害以及下半年国内外复杂多变的经济形势影响，抢抓机遇，开拓创新，扎实工作，全力推进经济社会快速协调发展，全市经济社会建设取得了历史性的突破。

初步预计：

——地区生产总值实现410亿元，增长14%左右；财政收入完成29.08亿元，增长26.4%；全社会固定资产投资完成203亿元，增长30%。

——三次产业的比重调整为24.6：40.3：35.1，二产比重提高了1.5个百分点，工业化水平达35.7%，比上年提高1.7个百分点，工业的主导地位进一步显现。

——外贸进出口总额1.65亿美元，增长44%;实际利用外资1.33亿美元，增长40%。

——城镇居民人均可支配收入12859元，增长20%；农民人均纯收入4003元，增长15.3%；三届人大四次会议确定的为民办十件实事基本完成。

——城镇化率达到33.3%,比上年提高2.2个百分点。

——节能减排工作取得良好成效，基本实现年度工作目标。

——推进信用体系建设取得显著成绩，金融环境得到较大改善，荣获“中国金融生态城市”称号。

——维稳工作成效显著，在全国“两会”、北京奥运会、第五届中国——东盟博览会、自治区成立50周年大庆等几个重大活动期间实现平稳安定，得到了自治区的充分肯定。

一年来，我们主要做了以下几方面工作：

（一）积极应对金融危机，国民经济实现平稳较快发展。上半年我市经济社会发展势头很好，但下半年以来，国际经济环境急转直下，国内经济困难明显增加，我市经济随着金融危机蔓延而出现了下滑态势。根据形势发展变化，我们冷静分析，采取召开传达学习中央宏观政策报告会和当前形势报告会、利用宣传媒体开辟专栏营造应对危机加快发展的舆论、举办专题论坛、召开重点行业业主座谈会、深入企业调查研究、出台切合本市实际的促进投资扩大内需的方法、财政扶持困难企业等一系列新举措，使全市广大干部群众较好地认清了当前的新形势，增强了应对困难、促进增长的信心，全市经济在不利因素增多的情况下仍然实现较快增长。工业方面，通过重点打造“五大产业基地”，大力发展“十大骨干企业”，建材、电力、糖纸、饲料、冶金、机械制造、农产品加工等支柱产业得到较快发展，产业集群优势逐步形成。开展了“百万标准厂房”大会战，园区实际可用面积扩大到20多平方公里，园区的承载能力和集聚效应进一步凸现。工业实现增加值146亿元，增长23%。招商引资上项目方面，通过开展招商引资项目“大兑现”活动，投资环境得到了较大改善，承接产业转移工作成效显著，新引进合同项目111个，实际利用市外资金100.1亿元，增长24.1%。非公有制经济快速发展，年内新增民营企业838家，民营企业实现工业增加值110亿元，占全部工业增加值的84%。农业方面，农业产业化步伐加快，优势产业和特色种养业规模进一步扩大，林业产业加快发展,农机科技推广工作成效显著，乡镇兽医站扩建改造任务基本完成。农林牧渔业总产值完成172.3亿元，增长5.8%；粮食总产量达135.9万吨，增长1.37%，实现大灾之年增产增收。第三产业方面，交通和商贸业发展势头喜人，港口货物吞吐量达3112万吨、集装箱吞吐量达5.04万标箱，分别增长24.14%和200.38%。北京华联、南宁百货、银座华隆百货、吉田超市等大型商贸流通企业进驻贵港，农村商贸流通网络初步形成,全社会消费品零售总额达151亿元，增长22%。旅游接待总人数和总收入分别增长12.5%和30%。

（二）坚持扩内需促投资，城乡基础设施建设步伐加快。认真贯彻落实中央和自治区关于加快基础设施建设、拉动内需的部署，着力抓好城乡基础设施建设。按照“城市框架向南北扩展、产业布局向东西延伸”的思路，进一步加强市中心城区的规划和建设，开展了城市总体规划修编工作。筹资4.28亿元投入城市基础设施建设，迎宾大道北延段、荷城路西延段建设基本完成，城西污水处理厂、垃圾处理厂建设步伐加快，“一路二中心三馆五园”建设进展顺利。房地产项目建设扎实推进，年内重点建设的39幢高楼已有16幢封顶。深入实施“城乡清洁工程”，城乡环境进一步美化。按照自治区打造西江亿吨黄金水道的战略部署，以建设现代化港口为目标，努力实施“港口带动”战略，筹资8.27亿元加强了港口基础设施建设，港口的竞争力和可持续发展能力显著提高，已成为全国十大内河港口之一。城乡路网、电网、水利等基础设施建设步伐加快，投入2.76亿元用于城乡路网建设，武宣至平南二级公路（贵港段）实现竣工，凤凰至覃塘二级公路、平南至金秀二级公路加快推进；投入4.2亿元加强城乡电网项目建设，年内有14个电力线路工程和变电工程实现竣工投产；投入3.58亿元，对34座水库实施了除险加固，对达开、武思江、六陈等大型灌区实施了续建与节水工程，并加快了贵港、桂平、平南等三个城区的防洪工程建设。

（三）着力解决民生问题，人民群众得到更多实惠。我们始终不渝地把为人民群众谋利益作为政府工作的着力点和落脚点，让广大人民群众共享改革发展成果。社会保障体系进一步完善，年内新增就业人数14701人，下岗失业人员再就业4223人，城镇登记失业率为3.4%；投入2016.8万元救助灾民35.7万人；支出保障金1.18亿元，保障了低保对象20.8万人。扶贫开发工作扎实推进，减少绝对贫困人口和低收入人口2.2万人。认真落实了年初确定的10件为民实事：新型农村合作医疗县市区覆盖率达到100%，参合率达到85.2%；发放救助资金386.12万元资助了4249名新入学的贫困大学生；发放2938.8万元资助了3.76万人次在市内中等职业学校就读的学生；免费为850名贫困家庭残疾儿童作矫治康复治疗；扶持农户新建沼气池6100座；投入4726万元新建人饮工程67项，解决了14.7万农村人口饮水难和饮水不安全的问题；筹资4.26亿元，开工建设村屯公路115条508.9公里，全市通公路行政村比例达到96%，通水泥路的行政村比例达到50%;农民实用技术和转移就业技能培训人数达40万人次；投入800多万元在中心城区改造了123条小街小巷，新建了13座公厕和5座垃圾中转站，硬化了48个连接城区主次干道出入口路面；筹资9650万元建设了5万平方米经济适用房；投入370万元新建和扩建了10个乡镇敬老院；在中心城区的新区规划定点了一所综合医院和一所初中。这些民生工程的实施，受到了市民的普遍赞誉。

（四）坚持统筹兼顾，各项社会事业协调发展。认真按照科学发展观的要求，高度重视发展各项社会事业，加大财政向社会事业领域投入和转移力度，全年用于科技、教育、文化、卫生、体育等社会事业的财政支出达17.2亿元，比上年增长32.7％。启动实施第四轮科技创新计划，取得科技成果34项，研发工业新产品40项，授权专利53件，获得自治区科技进步三等奖2项。“两基”成果继续得到巩固和提高，职业教育攻坚全面实施，其他各级各类教育取得显著成绩。文化事业发展加快，文化基础设施建设不断加强。群众性体育活动蓬勃开展，体育竞技水平得到提升。卫生医疗设施进一步改善，在市中心城区建立了10个社区公共卫生服务中心，公共卫生服务能力得到提高。广播电视“村村通”工程建设加快。精神文明建设扎实推进，文明城市、文明单位、文明村镇创建活动取得显著成效，创建卫生城市已通过自治区复审。人口和计划生育工作取得新成绩，人口自然增长率控制在9.5‰以内。环保工作得到加强，生态环境不断改善。建立了突发事件应急管理机制，社会治安综合治理和社会矛盾纠纷化解工作成效显著，各种刑事犯罪行为受到严厉打击，安全生产措施得到较好落实，年内没有发生重特大安全生产事故。其他各项社会事业也得到全面发展。在统筹我市各项社会事业发展的同时，我们坚持以党和国家大局为重，主动为国家分忧，统筹落实好上级下达的事关国家安全稳定的重要任务。尤其是四川特大地震发生后，我们以高度的责任感，及时组织捐款2135.3万元支援灾区，还从公安、消防、卫生等部门抽调人员奔赴四川灾区，出色地完成了现场抢救和震后服务的各项任务。

（五）强化机关作风建设，政府施政能力进一步提高。根据新形势新任务的需要，着力推进机关作风效能建设长效管理，全体公务员的服务意识进一步增强，工作效率得到提高。认真开展继续解放思想大讨论活动，广大干部思想进一步解放，创新意识进一步增强。加强了政府机关“依法行政规范化，依规办事标准化”的“两化”建设，修订了政府工作规则，全面落实了首问负责制、限时办结制和责任追究制的“三项制度”，进一步完善了行政运行机制，规范了行政执法行为，政府职能得到更好发挥。加强了政务信息网络建设，行政效率进一步提高。加大了政务公开力度，努力构建惩治和预防腐败体系，行政监察和纠风工作得到加强。注重解决群众反映的热点难点问题，查处了一批损害群众利益的案件。

各位代表，过去的一年是我市发展史上很不寻常、很不平凡的一年，也是改革和发展成效最好、基础打得最牢、人民群众得到实惠最多的一年，成绩来之不易。这是自治区党委、政府和市委科学决策、正确领导的结果，是市人大、市政协和社会各界监督支持的结果，是全市各族人民团结拼搏的结果。在此，我代表市人民政府，向全市广大干部群众，向各民主党派、工商联、无党派人士和社会团体，向驻贵部队官兵、公安民警和中区直驻贵单位干部职工，向关心支持贵港发展的社会各界人士，表示衷心的感谢和崇高的敬意！

回顾一年来的工作，我们也清醒地看到，我市在发展过程中还存在许多困难和问题，主要表现在：受国际金融危机影响，我市实体经济普遍受到冲击，下半年多数企业的生产下滑，税利减少，尤其是出口企业订单减少、市场萎缩、经营十分困难；农业基础设施还比较落后，农村保障体系比较薄弱，农民持续增收难度加大；转变发展方式、加快结构调整和节能减排的任务十分繁重；财政总量偏少、收支矛盾突出、公共财政覆盖面还不够广、收入差距大的问题仍然没有得到很好解决；各级领导对当前这场难以预料的国际金融危机还缺乏足够的认识，应对危机和防范风险的能力还需进一步提高。对这些在前进中出现的困难和问题，我们将客观分析、正确对待、千方百计予以解决，为实现平稳较快发展目标奠定更加坚实的基础。

2009年工作任务

2009年是新中国成立60周年，也是推进“十一五”规划顺利实施、实现我市科学发展三年计划的关键之年。做好今年经济社会发展工作，对于有效应对国际金融危机冲击、加快富裕文明和谐贵港建设步伐至关重要。

当前，由美国次贷危机引发的金融危机愈演愈烈，迅速从局部发展到全球，从发达国家传导到新兴市场国家和发展中国家，从金融领域扩散到实体经济领域。目前这场金融危机不仅本身尚未见底，而且对实体经济的影响正进一步加深，其严重后果还会进一步显现。尽管我国经济发展面临着来自国际国内的严峻挑战，但我国经济发展的基本面没有改变，重要战略机遇期仍然存在。受国际金融危机快速蔓延和世界经济增长明显减速的影响，我市和全国各地一样，经济运行中的困难增加，经济下行压力加大，经济生活中的深层次矛盾还将在今年凸现，年内更需要我们做好过紧日子的充分准备。虽然我们遇到了前所未有的困难，但也蕴含着很多加快发展的有利条件，机遇大于挑战，特别是国家近期出台的扩大内需促进增长的政策，必将给我市带来加快发展的巨大空间。另外，经过建市10多年的建设和积累，我市发展的基础日渐厚实，自我发展的能力明显增强，加上我们拥有资源、区位和政策等三大优势，发展的潜力还很大。因此，只要我们认真分析和准确把握国际国内经济走势，充分利用好国家宏观调控的各种支持性政策，审时度势，居安思危，深谋远虑，科学决策，坚定信心，扎实工作，完全有条件变压力为动力，化挑战为机遇，推动经济社会又好又快发展。

基于当前国内外复杂多变的经济形势，今年我市政府工作的总体要求是：以邓小平理论和“三个代表”重要思想为指导，深入贯彻科学发展观，全面落实党的十七大和十七届三中全会精神，紧紧围绕科学发展三年计划，按照“六靠”工作思路，突出“快速、科学”，立足扩大内需保持经济平稳较快增长，加快调整经济结构和转变发展方式，积极推进改革开放和自主创新，着力加强资源节约和环境保护，切实改善民生促进社会和谐，推进经济社会科学发展、和谐发展、跨越发展。

今年经济社会发展的主要预期目标为：全市地区生产总值增长12.5%，财政收入增长13%，全社会固定资产投资增长20%，社会消费品零售总额增长18%，城镇居民人均可支配收入增长10 %，农民人均纯收入增长8%，居民消费价格指数控制在5%以内，单位生产总值能耗下降3%，化学需氧量和二氧化硫排放量分别削减8%、7%；城镇登记失业率控制在4%以内，人口自然增长率控制在10‰以内。 在实际工作中，我们将遵循能快不慢、又好又快的原则，适时调整，力争超过预期目标。

实现上述预定目标，重点要抓好以下工作：

（一）加快推进农村改革发展，努力增加农民收入

深入贯彻落实党的十七届三中全会和中央农村工作会议精神，更加重视“三农”工作，加大“三农”投入，加强农业基础，增加农民收入，保障农民权益，促进农村和谐。

积极发展现代农业。以城乡一体、富裕农民为目标，认真落实支持粮食、生猪发展等各项政策措施，保护农民的生产积极性。在确保粮食播种面积和产量的基础上，坚持以市场为导向，大力发展食用菌、木薯、水果、中药材、蚕桑、花卉、蔬菜、林木、生猪、家禽、名优水产品等优势产业，力争在扩大规模、提升档次、增加效益上有新突破，进一步推动农业优势产业向规模化、集约化、标准化发展。按照“一个产业抓一两个核心企业、培育一两个优势品牌”的思路，整合涉农财政资金，落实扶持政策，集中扶持发展中丝茧丝绸、暨阳丝绸、扬翔公司、温氏集团、巨东集团、大福穗米业、金源酒精、三桂食品等一批农业产业化龙头企业，加快推进落实天福集团、百菇园等一批农业产业化项目，培养一批市场占有率高的知名农业品牌。继续扶持发展畜牧水产业，扩大以黄沙鳖、乳鸽等为重点的特色养殖业规模。支持农村科技创新和成果转化，完善农业科技推广体系，继续实施科技下乡、科技特派员、科技扶持工程。认真扶持和发展农民专业合作组织，年内新组建130个以上农民专业合作组织。

努力拓宽农民增收渠道。把提高农民收入、夯实农业基础作为扩大内需的重要内容，在大力发展农村二、三产业的同时，引导农民有序外出就业。针对当前外出务工农民工集中返乡的新情况，采取多种措施扶持返乡农民工就业和创业。鼓励企业使用本地农村劳动力，拓展公益性岗位，促进农村劳动力充分就业。加强农村实用技术培训和农村劳动力转移就业工作，年内完成各类培训43万人次以上，实现农村劳动力转移就业90万人以上。实施第三批整村推进扶贫工作，因地制宜重点扶持29个贫困村，帮助贫困地区群众尽快摆脱贫困。

加强农村基础设施建设。抓住国家扩大农村内需的机遇，努力增加农村基础设施建设和社会事业发展的投入。计划开工建设通乡水泥路5条74公里，修建通达工程项目49个232公里、通村水泥路50条200公里，争取提前两年实现“村村通公路”目标。建好一批农村客运站场。年内筹资5亿元加强农村水利设施建设，加快建设市中心城区郁江北堤、郁江南堤和桂平防洪堤建设；新建人饮安全项目95项，解决农村25万人饮水困难和饮水不安全问题；全面完成20座水库除险加固任务，继续抓好达开、武思江、六陈等大型水库灌区节水改造项目，完成硬化主渠道35公里，新增恢复有效灌溉面积2万亩，改善灌溉面积10万亩。加快农村改水、改厕、改厨、改栏步伐，推广农村清洁能源，新建沼气池12000个。抓好农村电网改造，改善农村通信条件。因地制宜抓好社会主义新农村示范村的规划和建设。

认真落实各项惠农政策。严格落实粮食最低收购价、各项农业补贴、水库移民后期扶持等惠农政策。扩大公共财政覆盖农村范围，进一步增加农村教育、文化、卫生、社会救助等投入，促进农村公共事业发展。认真执行减负政策，严格涉农价费监管，规范“一事一议”，切实减轻农民负担。积极发展农村小额信贷。加强农业执法和农资市场管理，切实维护农民利益。

深化农村综合改革。稳定和完善农村基本经营制度，按照依法自愿有偿的原则，引导农村土地经营权流转，促进土地适度规模经营。进一步深化乡镇机构改革、农村义务教育管理体制改革、县乡财政管理体制改革，统筹推进农村金融体制、户籍制度、征地制度、集体林权和水权制度等改革，促进城乡经济社会协调发展。

（二）强化产业支撑，壮大工业经济实力

工业化是贵港的主导方向和首要选择，是全市经济发展的主战场。要密切关注经济运行环境的复杂性、多变性和国家宏观调控政策的导向，更加注重工业结构调整和产业升级，更加注重企业的自主创新能力，真正实现工业经济好中求快、优中求进，全面提升我市工业综合实力和竞争力，实现全部工业总产值突破600亿元，规模以上工业企业产值比增35%以上、增加值增长28%以上。

发展壮大优势产业。主动适应市场经济规律，围绕培植壮大建材、电力、冶金、糖纸、化工等传统支柱产业，制订新的激励措施扶持骨干企业和纳税大户，在用地、用工、科技、信息、技改资金等方面向骨干企业倾斜，缓解技术改造、结构调整、争创品牌、技术创新、节能降耗、高新技术开发研究等方面的资金制约，促进企业提速提质，扩大生产，提高效益，力争全年实现销售收入超亿元的企业50家以上，其中超10亿元企业9家以上，超5亿元企业16家以上；税收超1亿元企业7家以上，超5000万元企业15家以上。建立健全中小企业服务体系，加大扶持力度，促进中小企业不断提高技术和管理水平，增强发展活力与市场竞争力。引进一批战略合作伙伴，力争新增规模以上工业企业85家以上，新增亿元工业企业10家以上。同时，实施精品名牌战略，培育发展一批中国名牌、中国驰名商标和地方名牌。

更加注重经济运行协调。针对当前国内外经济形势的发展变化，进一步增强应对危机和适应宏观调控的预见性、针对性、有效性，建立健全“企业为主、政府推动、部门配合”的工作机制，加大经济运行协调力度，制定和出台帮助企业解困的新办法，在融资贴息、产品营销、运输调度、劳动保障等方面，优先扶持重点产业和重点企业，确保工业经济平稳较快发展。与此同时，加强区域经济发展研究和协调，通过制定规划、举办专题论坛、加强与沿江城市合作等方式，努力构建沿江经济带，促进沿江工业合理布局，并依托西江“黄金水道”迅速崛起，成为推动经济又好又快发展的巨大动力。

抓好重大工业项目。切实把握国家投资导向，抓好117个投资额在1000万元以上的新开工项目，突出抓好156个投资额在5000万元以上的在建项目，重点推进平南纸浆、桂平华润、远辰锰业、覃塘鑫发冶炼等一批重大项目开工建设和清隆机械、镁星镁业、史丹利复合肥等一批重大续建项目，争取尽早投产见效。年内计划投资50亿元，完成贵钢技改、金源酒精技改、宝马铁合金技改等90个重大项目的技改任务。千方百计协调好各方面关系，抓好高铁三水铝、粤港皮革环保工业城和桂平陶瓷基地建设，力争通过三至五年努力，将贵港打造成为广西乃至全国有名的铝业、皮革和陶瓷产业基地。积极推进平南华润四期、台泥三期、华电二期等一批重大工业项目的前期工作。坚持实行重点项目倒排工期、跟踪督查、联席会议和目标考核四项制度，在重点项目规划、用地、建设等方面，做到急事急办、特事特办，提供优质高效服务。

发展壮大园区经济。进一步明确园区的产业定位和方向，突出发展特色优势产业，把工业园区作为承接产业转移的主要基地，实现产业集群、要素集聚、服务集成。重点加快推进市江南工业园三期、西江产业园一期和覃塘区石卡临江工业园区的基础设施建设，规划建设平南新桥工业集中区，扩大桂平长安工业园、龙门食品工业集中区、木乐服装工业集中区的规模。启动建设市江南工业园污水处理厂。改革创新园区管理体制和运行机制，着力完善信息、金融、保险、技术、仓储、物流、厂房租赁、法律咨询等后勤保障服务，不断增强产业承载功能。加强土地储备，加快园区基础设施建设，全年建设工业标准厂房30 万平方米以上，为企业入驻创造更好的条件。紧紧抓住东部地区外需减少、产业急剧转移的重大机遇，坚持以空间换时间、以存量换增量、以资源换项目，实施招商引资项目“大兑现”和“百万标准厂房招商引资”活动，积极引进和新上一批符合国家产业政策的工业项目。力争全年引进市外资金125亿元，合同项目履约率超过95%、开竣工率超过55%、资金到位率超过35%以上。

提高企业创新能力。抓住国家出台政策加快结构调整、鼓励企业加大固定资产投入的机遇，围绕增强企业综合竞争力和应对市场风险的能力，进一步加强以企业技术中心为主体的技术创新体系建设，增强重点产业和骨干企业的自主研发能力，加快产品和技术引进消化吸收的再创新。通过政策扶持，提高企业技改的能动性，引导企业积极与市外企业合作，推进产业配套，尽快扭转目前存在的产业链条短、产品附加值低的状况。着力推动产学研合作，积极争取国家和自治区科技项目，促进科技成果与产业发展相结合，提高产业科技水平。加大信息化与工业化的融合力度，积极推进以完善骨干企业信息化应用与管理为重点的信息化项目建设。力争新增自治区级以上工程中心或技术中心1家以上；新增高新技术产品2—3个，1—2家企业通过国家新的高新技术企业认定办法认定，高新技术产业产值增长20%以上。扶持民营科技型企业发展10家以上，企业开发新产品40个以上，新产品产值增加10%以上。

（三）千方百计抓投入促消费，努力扩大内需对经济发展的拉动力

把扩大内需作为保增长的根本途径，把保持投资增速与优化投资结构有机结合起来，努力争取国家项目和资金，加快项目建设，拓展城乡消费，优化出口结构，进一步增强经济发展后劲。

狠抓基础设施和基础产业重点项目建设。全面把握国家宏观调控区别对待、有保有压的原则，优化投资结构，谋划建设一批符合国家政策、具有比较优势的重大项目，保持投资对经济增长的持续拉动。重点抓好产业和基础设施建设投资额超千万元的项目147个，年内计划完成投资额120.2亿元。抢抓机遇，全力协助国家开工建设南广高速铁路和贵港至梧州高速公路，建成覃塘至凤凰、平南至金秀二级公路，配合做好贵港至来宾高速公路建设的前期工作，力争木格至水鸣、山心至大圩、港南至平山的二级公路列入自治区路网建设规划，进一步改善交通环境。加快建设污水处理厂和城市生活垃圾处理厂。紧紧抓住自治区决定打造“西江亿吨黄金水道”的重大契机，加快猫儿山作业区二期工程、贵港至梧州二级航道、桂平航运枢纽二线船闸、台泥公司专用码头和平南武林港二期等工程建设，谋划建设中心港区扩建工程，整合提升沿江港口功能，更好地实施江海联运，努力实现港口货物吞吐量3500万吨、集装箱6万标准箱以上。开工建设大藤峡水利枢纽工程，加快推进白沙核电项目的前期工作。继续加强项目库建设，开发储备一批大项目、好项目。

努力促进城乡消费。采取强有力的措施，进一步提高第三产业比重，增强消费需求对经济增长的拉动作用。合理引导住房、文化、健身、娱乐、休闲等消费，大力发展交通运输、现代物流等生产性服务业，培育新的消费热点，促进消费结构升级，以消费结构升级带动产业结构调整。支持建设景泰物流、中联物流、海楼物流、永泰仓储物流、港龙物流等现代物流项目，培育一批大型专业批发市场，增强市场辐射功能和拉动效应。加快适应需求的商品房建设，改进和规范经济适用住房制度，构建多层次住房保障体系，增强居民消费预期。抓住国家对农民购买家电给予补贴的机遇，着力促进农村消费，扩大农村内需。深入实施“万村千乡市场工程”，开展“工业品下乡农产品进城促销活动”，健全农产品进城、工业品下乡的流通体系。引导和促进各类投资主体到农村投资建设商业设施，鼓励各类连锁超市向农村延伸，发展一批现代连锁农家店，更好地释放农村消费潜能。充分发挥我市人文历史资源优势，以创建新的4A级景区为重点，加强旅游基础设施建设，抓好旅游产品开发和促销，做强做大旅游产业，促进旅游消费增长。提高城乡居民特别是农民和城镇低收入居民的收入水平，正确引导消费需求，不断拓宽消费领域。

扩大对外经济贸易。积极适应国家外贸政策调整的新变化，巩固发展原有出口基地，优化出口产品结构，培育新的出口增长点。特别是要紧紧抓住发达地区外贸出口调整出现的空档，有针对性地扶持出口企业调整产品结构、提高竞争力，适时拓展国内外市场，保持进出口稳定增长。

扶持非公有制经济加快发展。进一步放宽市场准入，加大扶持力度，营造宽松环境，鼓励非公有制经济参与国有和城镇集体企业产权制度改革，参与城市基础设施和市政公用设施建设和营运，参与农业综合开发。引导商业银行加大对非公有制企业的信贷服务，拓宽投融资渠道，重点扶持比较优势明显、带动作用强的龙头企业，引导非公有制企业走规模型、科技型、外向型的发展道路，努力实现非公有制经济发展的新突破。

（四）着力提升城市品位，加快推进城镇化进程

紧紧围绕建设“区域性中心城市和南国特色园林现代化港口城市”的目标，大力实施品牌城市战略，不断创新城市发展理念和模式，全面提升城市综合素质和竞争力，力争城镇化率提高2个百分点，达到35.3%。

完善城市规划。认真贯彻《城乡规划法》，充分发挥城市规划的龙头作用，进一步加强城市规划工作，高标准完成城市总体规划，搞好城北新区二期、江南片区等城市规划区控制性详细规划、修建性详细规划和专业规划。高质量完成第二次土地利用总体规划修编，调整优化土地利用结构和布局。加大城市规划执行力度，严肃查处违法占地、违规建设等行为，切实维护城市规划的法定性、权威性和严肃性。

加强城市建设与管理。坚持把城市建设作为拉动投资和内需的重要渠道，加快城建重点项目建设，拉大城市框架，完善城市功能，提高城市档次和品位。重点推进东湖综合整治工程，继续抓好中山北路、迎宾路北延段、荷城路西延段工程建设，开工建设布山路、桂林路、建设西路延长线、人民路、新区三路和郁江南堤路园以及文化艺术中心、体育中心、博物馆、图书馆、档案馆，启动建设南湖公园、民族文化公园等景区基础设施。马草江公园、南山公园实现竣工开园。完成市中心城区垃圾处理厂和生活污水处理厂的配套设施建设，改造中心城区小街小巷200条，新建20座公厕、5座垃圾压缩中转站。在新区开工建设荷城初中，筹备新建一所综合性医院。以创建国家园林城市为目标，深入实施“城乡清洁工程”，加强城市综合管理，确保市容整洁有序，绿化、亮化、美化水平有新提高，发展环境和人居环境进一步优化。加强土地储备工作，依法严格管控土地一级市场，为加快城市发展提供土地保障。

促进房地产业持续健康发展。认真落实国家出台的房地产政策，从土地、金融、税收、行业管理等方面加大对房地产企业的扶持，提高企业抗风险能力。充分发挥市场配置土地资源的基础作用，最大限度地节约集约利用土地。加快建设在建住宅楼盘，适度推进高层建筑建设，力求做到单体设计出精品、整体建设出形象，打造城市新亮点。正确运用政府调控和市场机制两个手段，保持房地产投资合理规模，重点发展面向广大群众的普通商品住房。加强房价监管和调控，规范房地产市场秩序，依法惩治房地产开发、交易、中介等环节的违法违规行为。

（五）抓好财税金融工作，增强财政保障能力

综合分析影响2009年经济形势和财政收支的各种因素，预计今年将是财政收入比较困难的一年。为此，我们要审时度势，未雨绸缪，按照促进增长、调整结构、推动改革、注重民生的原则，加强财税工作和金融监管，构建保障有力的公共财政服务体系。

努力促进财源稳定增长。针对国家全面实施增值税改革、资源税改革以及税费政策调整的新情况，积极发挥财政职能作用，充分运用贴息、补贴、补助、奖励等财政税收杠杆，扶持税收主体和骨干税源，支持重点财源项目加快建设和发展，培植一批税收超千万元的纳税大户，促进税收持续较快增长, 努力提高税收占财政收入的比重。坚持依法治税，科学征管，打击各种涉税违法行为，确保应收尽收。规范非税收入征缴和管理，拓宽财政增收渠道。

加强财政预算管理。今年要按照控制非生产性支出，增加扩大内需、促进经济增长支出的原则，调整优化支出结构，增加社会保障、教育、卫生等方面支出，加大对农业和农村发展、自主创新、能源资源节约、生态环保以及主导产业等重点领域的投入，保障经济社会发展薄弱环节和公共服务支出需要。严格控制一般性支出，对公务购车用车、会议经费、公务招待费用、出国（境）经费等支出实行零增长，减少行政成本。严肃财经纪律，严格执行国库集中收付和政府采购制度、财政投资评审制度，推行公务卡结算方式，加强公务支出资金的监管，提高资金使用效益。规范行政事业单位占有、使用、处置国有资产行为，加强政府债务管理，提高科学理财水平。

进一步发挥金融对经济的支撑作用。正确把握和用足用活国家实施适度宽松的货币政策，充分发挥获得“中国生态金融城市”称号的效应，更加重视社会信用环境建设，加强政银企合作。鼓励和支持商业银行拓宽服务领域，增加信贷投放量，特别是对重点项目、重点企业的信贷投入，创建银企双赢的发展平台。加强中小企业信用体系建设，建立融资、担保平台，更多地吸纳各金融机构的资金，增强对城市基础设施、中小企业和民营企业的扶持。加强农村信用体系建设，引导金融机构加大对“三农”的信贷支持。

（六）坚持以人为本，努力构建和谐社会

高度重视和解决民生问题，对原有的惠民政策要按照规定扩大覆盖范围，提高相应标准；对新出台的惠民政策要加大落实力度，多为群众办实事，让人民群众得到更多的实惠。

实施积极的就业政策。开展全民创新创业活动，健全创业服务体系，完善支持自主创业和自谋职业的各项政策，促进以创业带动就业。增加公益性岗位，积极帮助下岗失业人员再就业、“零就业”家庭和就业困难人员就业。加强对高校毕业生就业的指导和服务，高度重视退伍转业军人、残疾人就业问题。加强对城镇下岗失业人员再就业、农民工技能和农村中学毕业生的培训，进一步增强城乡劳动者就业和创业技能。落实信贷融资支持和相关税费减免政策，出台相关补贴和激励措施，鼓励中小企业吸纳更多的劳动力，为城乡居民提供更多的就业岗位。加强城市社区建设，积极开辟社区服务新项目，减轻就业再就业的压力。年内计划城镇新增就业1.2万人以上，下岗失业人员再就业3000人以上，新增农村转移就业3.8万人以上。

完善社会保障体系。积极扩大城镇社会保险覆盖面，尽快使养老、医疗、失业、工伤和生育等社会保险覆盖到各类企业。加快推进事业单位养老保险制度改革，探索建立农村养老保险制度和政策性农业保险。实施城镇居民基本医疗保险和被征地农民社会保障工作，扩大失业保险覆盖面。深入推进新型农村合作医疗制度，不断提高参合率。规范农民工劳动管理制度，积极探索适合农民工特点的工伤、医疗、养老等社会保障制度。继续完善城乡低保、城乡医疗救助制度。加强农村敬老院建设。发展残疾人事业。加快经济适用房和廉租住房建设，认真兑现补贴政策，着力解决城市低收入家庭住房困难。加强物价监管，保持市场物价基本稳定，保障市场供应。

统筹发展各项社会事业。实施第四轮科技创新计划，加强创新型人才和各类紧缺人才的培养、选拔和引进，促进人才资源的合理配置。优先发展教育事业，巩固和提高义务教育，扎实推进职业教育攻坚，完成职业教育攻坚目标任务，规范和鼓励发展民办教育，促进各级各类教育协调发展。增加农村义务教育公用经费，加快农村中小学校舍维修改造，加强教育信息化建设，进一步改善农村办学条件，促进教育均衡发展。大力发展体育事业，加强城乡社区体育设施建设。抓好城乡基层文化设施建设，力争年内新建33个文化站和100个村文化活动室。继续抓好广播电视“村村通”工程，力争年内完成20户以上通电自然屯“村村通”建设任务。稳步推进医药卫生体制改革，加快建立城市社区卫生服务网络，加强农村三级卫生服务网络建设。全面落实人口计生奖励扶助政策，加强流动人口计生管理，稳定低生育水平，提高出生人口素质。深入开展双拥共建活动，增强军政军民团结。充分发挥工会、共青团、妇联等群团组织的作用。统筹抓好其他各项社会事业。组织力量做好‘十二五’规划的前期工作。

抓好节能减排工作。严格实行节能减排问责制和“一票否决”制，打好节能减排攻坚战。严格执行重点产品能耗限额标准，加强高耗能行业节能管理，淘汰落后生产能力，依法关闭破坏资源和污染环境的企业。加强对新建项目的节能环保准入审查。推广多层标准厂房，提高土地集约利用水平。推进农村、建筑行业、交通运输、政府机构等领域的节能工作。继续抓好重点区域和行业的环境综合整治，搞好环境保护。加快建立完善城乡垃圾收集清运处理系统。控制农村面源污染。加强生态环境保护和中心城区第二水源保护，实现可持续发展，造福全市民众。

加强社会管理。创新社会管理体制机制，充分发挥各类社会组织在构建和谐社会中的积极作用。进一步落实安全生产责任制，狠抓各项措施的落实和安全隐患整治，杜绝重特大事故发生。加强信访工作，及时化解各类社会矛盾。加强精神文明建设，深入开展平安创建活动，健全社会治安防控体系，依法打击各种违法犯罪行为，教育广大群众远离“六合彩”和各种形式的赌博，自觉抵制社会丑恶现象。强化市场监管，确保产品质量和食品药品安全。进一步完善公共应急体系建设，积极预防和妥善处置各类突发事件，维护社会和谐稳定。

（七）提高领导科学发展水平，努力开创各项工作新局面

主动适应改革开放和现代化建设的新形势，着力推动政府自身改革和建设，为加快发展提供思想和能力保障。

注重学习，提高科学判断形势的能力。面对当前复杂多变的国内外形势，引导各级政府机关干部不断加强学习。既要学习政治理论和政策法规，也要学习历史、科技、经济等方面的知识；既要掌握国情、区情、市情，也要懂得世界动态，使广大干部都善于分析形势、把握大局、趋利避害、战胜困难，夺取胜利。

开拓进取，提高务实创新的能力。引导各级各部门自觉将贵港的发展放在全区乃至全国发展的大背景下去把握、去谋划，在全面贯彻落实国家宏观调控政策的同时，认真研究加快贵港发展的新思路、新方法，牢牢掌握工作的主动权。大力弘扬敢于负责、善于创新、奋勇争先的开拓精神。坚持解放思想、实事求是、与时俱进，在加快发展中敢闯敢试、敢抓敢管、敢做敢为，以发展为己任，看准的事盯紧抓实，探索中的事勇于突破，有争议的事大胆尝试，用好、用足、用活各项支持发展的政策，加快发展步伐。

把握全局，提高应对复杂局面的能力。随着改革的不断深化，显现出的社会矛盾和社会困难越来越多，越来越复杂。尤其在当前挑战和机遇并存的形势下，我们必须准确把握党的方针政策，深入调查研究，善于集中民智，积极探索实践，保证决策的科学性和正确性。分析任何一种矛盾，都要考虑前因后果、相关因素；解决任何一个矛盾，都要既对上级负责又对下级负责，既对现实负责又对长远负责，既对局部负责又对全局负责，防止处置不当，引发群体性事件和公共突发事件。

真抓实干，提高执行重大决策的能力。贯彻执行中央、自治区和市委、市政府的重大决策以及解决具体问题、化解具体矛盾的能力是政府各部门执行力的重要体现。对定下来的事抓住不放，把抓落实体现在工作成效上。坚决克服以文件落实文件，以会议落实会议，以讲话落实讲话的形式主义，一步一个脚印地推进工作，确保圆满完成市委、市政府确定的各项目标任务。健全和强化行政督查机制和内部督查制度，加大对重大决策、重点工作、主要目标的督促检查，做到事事有着落，件件有结果。

勤政为民，提高公共服务的能力。大力倡导和弘扬勤奋工作、奋发有为的敬业精神，营造扎实工作，恪尽职守，积极奉献的良好氛围。加快转变政府职能，在履行好“经济调节、市场监管”职能的同时，更加注重公共服务和社会管理职能，不断提高政府的公共服务能力。深化审批制度改革，提高行政效率，降低行政成本。加强机关效能建设，继续推行职能部门公开服务承诺制，强化效能督查和绩效考核，推动行风、政风进一步好转，营造便民、利民、重商、扶商的良好发展环境。落实《建立健全惩治和预防腐败体系2008—2012年工作规划》，推进惩治和预防腐败体系建设，从源头上防治腐败，促使各级领导干部自觉为人民掌好权、用好权，建设廉洁政府。

各位代表，实现今年各项既定目标，任务艰巨，意义重大，使命光荣。困难和挑战考验着我们，责任和使命激励着我们，全市人民在企盼着我们。让我们更加紧密地团结在以胡锦涛同志为总书记的党中央周围，高举中国特色社会主义伟大旗帜，坚持以邓小平理论和“三个代表”重要思想为指导，以科学发展观统领全局，认真贯彻落实十七届三中全会精神，坚定信心，迎难而上，扎实工作，加快富裕文明和谐贵港建设步伐，以优异成绩向新中国成立60周年献礼！
